# Recognition of Mycenasect.Amparoina sect. nov. (Mycenaceae, Agaricales), including four new species and revision of the limits of sect. Sacchariferae

**DOI:** 10.3897/mycokeys.52.34647

**Published:** 2019-05-16

**Authors:** Qin Na, Tolgor Bau

**Affiliations:** 1 Engineering Research Centre of Chinese Ministry of Education for Edible and Medicinal Fungi, Jilin Agricultural University, Changchun 130118, China Chinese Ministry of Education for Edible and Medicinal Fungi Changchun China

**Keywords:** Agarics, new taxon, systematics, taxonomy

## Abstract

Phylogenetic reconstruction revealed that *Mycena* stirps *Amparoina*, which is traditionally classified in sect. Sacchariferae, should be treated at section level. Section Amparoina is characterised by the presence or absence of cherocytes, the presence of acanthocysts and spinulose caulocystidia. Eight species referred to Mycenasect.Amparoina**sect. nov.** are recognised in China. Of these taxa, four new species classified in the new section are formally described: *M.bicystidiata***sp. nov.**, *M.griseotincta***sp. nov.**, *M.hygrophoroides***sp. nov.** and *M.miscanthi***sp. nov.** The new species are characterised by the absence of both cherocytes and a basal disc, along with the presence of acanthocysts on the pileus, spinulose cheilocystidia and caulocystidia. Descriptions of the new species, accompanied by illustrations of morphological characters and comparisons with closely related taxa, are provided. A multi-locus analysis utilising the ITS + nLSU + SSU regions was carried out using maximum likelihood and Bayesian Inference. A key to the 12 species of sect. Amparoina**sect. nov.** and sect. Sacchariferae that are found in China is provided.

## Introduction

The genus *Mycena* (Pers.) Roussel is characterised by small basidiomata, thin and convex pileus with sulcate margin, non-deliquescent lamellae and hollow stipe (Persoon 1797). The genus comprises more than 500 species and is distributed worldwide (Kirk et al. 2008). Mycenasect.Sacchariferae Kühner ex Singer, which is one of the largest sections in the genus, was first published as a *nomen nudum* by Kühner (1938), who defined the section to include members that possess a granulose or “sugar coated” pileus. In 1958, Singer erected the monotypic genus *Amparoina* Singer to house *Marasmiusspinosissimus* Singer based on the collections from Argentina (Singer 1958). Later, Singer (1976) established Amparoinaceae with *A.spinosissima* (Singer) Singer as type species and introduced another species in *Amparoina*, *A.heteracantha* Singer. Meanwhile he suggested that *Amparoina* was similar to sect. Sacchariferae, but maintained the autonomy of the former due to inamyloid basidiospores and revised sect. Sacchariferae to be characterised by a pileipellis with acanthocysts, which remain as terminal cells overlaid by a universal veil (Singer 1976). The pileus of cherocytes and acanthocysts distinguish taxa of sect. Sacchariferae from all other Mycena species. Section Sacchariferae was subdivided by Desjardin (1995) into stirps *Amparoina* Desjardin, stirps *Alphitophora* Desjardin and stirps *Adscendens* Desjardin, with 55 epithets classified into 27 taxa, based on presence or absence of a basal disc, cherocytes, and diverse caulocystidia. Maas Geesteranus and de Meijer (1997) established a fourth stirps, named stirps *Fuscinea* Maas Geest. & de Meijer, in which the acanthocysts possess brown contents, a character similar to that of stirps *Amparoina*. Only two species have been classified in stirps *Fuscinea*, namely *M.fuscinea* Maas Geest. & de Meijer and *M.fuliginea* Maas Geest. & de Meijer (Maas Geesteranus and de Meijer 1998). The morphology-based infrasectional classification of Mycenasect.Sacchariferae, proposed by Desjardin (1995), has been widely adopted. However, no phylogenetic reconstruction of relationships in sect. Sacchariferae has been published to assess the validity of the infrasectional classification.

Previous studies of sect. Sacchariferae have focused on species distributed in Europe and North and South America, with more than 60 species studied in the past 30 years (Maas Geesteranus 1983, 1992a, 1992b; Lodge 1988; Takahashi 1999; Perry 2002; Grgurinovic 2003; Robich 2003, 2016; Tanaka and Hongo 2003; Nealel 2009; Robich and Hausknecht 2009; Zamora and Català 2013; Cortéspérez et al. 2015; Aronsen and Læssøe 2016). In contrast, studies of Asian taxa have been scanty until recent years. Aravindakshan and Manimohan (2015) described ten taxa, including six new species in sect. Sacchariferae from India. Only three species, *M.anoectochili* L. Fan & S.X. Guo, *M.alphitophora* (Berk.) Sacc. and *M.cornephora* Maas Geest., were formerly reported from China (Guo et al. 1997; Li et al. 2015). However, recently, three new taxa of sect. Sacchariferae were described, namely *M.castaneicola* T. Bau & Q. Na, *M.hyalinostipitata* T. Bau & Q. Na and *M.substylobates* T. Bau & Q. Na, from subtropical regions of China (Na and Bau 2019).

A phylogenetic reconstruction of *Mycena* was incongruous with the traditional classification of stirps Amparoina within sect.Sacchariferae and indicated that the taxonomic classification of the section should be reconsidered. During our ongoing research on *Mycena*, four new taxa without a basal disc and cherocytes, belonging to the new section, were found in southern China in Chongqing City, Guangdong Province, Henan Province, Hubei Province, Tibet Autonomous Region, Yunnan Province and Zhejiang Province. These species are described here. Based on the phylogenetic analyses, an identification key to the 12 species of sect. Sacchariferae and sect. Amparoina currently known from China is provided.

## Materials and methods

### Morphological study

Macroscopic characters were described from fresh specimens following conventional taxonomic methods. Colour terms and notations refer to those of Kornerup and Wanscher (1978). Microscopic characters were observed from dried specimens rehydrated in 5% potassium hydroxide (KOH) and stained with Congo red, using a Nikon 80i light microscope. Melzer’s reagent was used for testing amyloid and dextrinoid reactions of all tissues (Horak 2005). The spore shape quotient (spore length divided by spore width; *Q* = *L*/*B*) was calculated from 40 mature basidiospores; 90% of the numerical range is indicated outside parentheses and the 10% extreme values are enclosed in parentheses. Author abbreviations are based on those used in Index Fungorum (https://www.indexfungorum.org). Voucher specimens have been deposited in the Herbarium Mycology of Jilin Agricultural University (HMJAU).

### DNA extraction, PCR amplification and DNA sequencing

Material for DNA isolation was taken from dried specimens. Genomic DNA was extracted from samples using the NuClean Plant Genomic DNA Kit (Kangwei Century Biotechnology Company Limited, Beijing, China). The internal transcribed spacer (ITS) region was amplified with the primer pair ITS1 and ITS4 (White et al. 1990). The nLSU and SSU regions were amplified using the primers LROR/LR7 and MS1/MS2, respectively (Ward et al. 1992; Hopple and Vilgalys 1999). The PCR cycling schedule for the ITS, nLSU and SSU region used a touchdown programme (Na and Bau 2018). All newly generated sequences were deposited in GenBank (Table 1).

**Table 1.** Sequenced specimens used in phylogenetic analysis.

Taxa	Voucher	Locality	GenBank accession no.		
			ITS	nLSU	SSU
*Infundibulicybegibba* (Pers.) Harmaja	AFTOL-ID 1508	USA	DQ490635	DQ457682	–
* I. gibba *	FLAS-F-60947	Unpublished	MH016906	–	–
*Mycenaabramsii* (Murrill) Murrill	HMJAU 43282	Jilin: Jingyuetan National Scenic Area, Changchun City	MH396626	MK629348	MK629326
* M. abramsii *	HMJAU 43468	Jilin: Jingyuetan National Scenic Area, Changchun City	MH396627	–	MK629328
	HMJAU 43523	Jilin: Songjiang Town, Jiaohe City	MH396628	MK629350	MK629330
	HMJAU 43606	Inner Mongolia Autonomous Region: Mangui Town, Hulunbeier City	MH396629	MK629355	MK629336
*M.adscendens* Maas Geest.	Orstadius329-05	Norway: Strengsdal Village, Vestfold	KT900141	–	–
* M. adscendens *	Aronsen061119	Norway: Strengsdal Village, Vestfold	KT900142	–	–
	Aronsen120826	Norway: Strengsdal Village, Vestfold	KT900143	–	–
* M. alphitophora *	HMJAU 43498	Jilin: Shenglihe forest farms, Jiaohe City	MH136830	–	MK629329
	HMJAU 43686	Yunnan: Zixi Mountain National Nature Reserve, Chuxiong City	MH136831	–	MK629343
*M.arcangeliana* Bres.	252b	Italy: Venice Museum of Natural History, Venice	JF908401	–	–
* M. arcangeliana *	252f	Italy: Venice Museum of Natural History, Venice	JF908402	–	–
*M.bicystidiata* T.Bau & Q.Na	HMJAU 43589	Hubei: Yandongwan, Lichuan County	MK309774	–	–
* M. bicystidiata *	HMJAU 43593	Hubei: Xingdou Mountain National Nature Reserves	MK309775	MK629354	–
	HMJAU 43648, **Type**	Chongqing: Dafengbao Scenic Regions, Huangshui Town	MK309773	MK629359	MK629341
	HMJAU 43744	Zhejiang: Tianmu Mountain National Nature Reserves, Hangzhou City	MK309776	–	–
*M.castaneicola* T.Bau & Q.Na	HMJAU 43578, **Type**	Henan: Jigong Mountain National Nature, Xinyang City	MH136826	–	MK629334
* M. castaneicola *	HMJAU 43581	Henan: Bolden National Forest Park, Xinyang City	MH136827	–	–
*M.citrinomarginata* Gillet	HMJAU 43563	Shanxi: Wutai Mountain National Nature, Xinzhou City	MG654739	MK629351	MK629331
* M. citrinomarginata *	317h	Italy: Venice Museum of Natural History, Venice	JF908416	–	–
	AD4TN	Tunisia: Aïn Draham	KU973883	–	–
*M.corynephora* Maas Geest.	HMJAU 43574	Henan: Xinyang City	MH136832	–	MK629332
* M. corynephora *	HMJAU 43576	Henan: Xinyang City	MH136833	–	MK629333
*M.diosma* Krieglst.&Schwöbel	320f	Italy: Venice Museum of Natural History, Venice	JF908417	–	–
*M.griseotincta* T.Bau & Q.Na	HMJAU 43800, **Type**	Yunnan: Shangri-La Pudacuo National Park	MK309783	MK629363	MK629346
* M. griseotincta *	HMJAU 43805	Yunnan: Shangri-La Pudacuo National Park	MK309782	–	–
	HMJAU 43819	Tibet: Zhuqudeng Village, Nyingchi City	MK309784	–	–
*M.heteracantha* (Singer) Desjardin	HMJAU 43709,	Hunan: Yuelu Mountain, Changsha City	MK309785	MK629362	MK629345
* M. heteracantha *	HMJAU 43711	Hunan: Xiaoxi National Nature Reserves	MK309786	–	–
	HMJAU 43716	Hunan: Gaowangjie National Nature Reserves	MK309787	–	–
*M.hyalinostipitata* T.Bau&Q. Na	HMJAU 43693, **Type**	Yunnan: Yeyahu Scenic Spot, Kunming City	MH136828	MK629361	MK629344
* M. hyalinostipitata *	HMJAU 43701	Yunnan: Yeyahu Scenic Spot, Kunming City	MH136829	–	–
* M. hygrophoroides *	HMJAU 43417, **Type**	Guangdong: Chebaling National Nature Reserve, Shaoguan City	MK309780	MK629349	MK629327
	HMJAU 43421	Guangdong: Shangxie Village, Shaoguan City	MK309781	–	–
*M.meliigena* (Berk.&Cooke) Sacc.	39	Italy: Venice Museum of Natural History, Venice	JF908423	–	–
* M. meliigena *	39d	Italy: Venice Museum of Natural History, Venice	JF908429	–	–
*M.miscanthi* T.Bau & Q.Na	HMJAU 43573	Henan: Jinniu Mountain, Xinyang City	MK309777	MK629352	–
* M. miscanthi *	HMJAU 43582	Henan: Bolden National Forest Park, Xinyang City	MK309778	–	–
	HMJAU 43584, **Type**	Henan: Jigong Mountain National Nature, Xinyang City	MK309779	MK629353	MK629335
*M.pearsoniana* Dennis ex Singer	FCME25817	USA: Great Smoky Mountains National Park, Tennessee	JN182198	–	–
* M. pearsoniana *	TENN61544	USA: Great Smoky Mountains National Park, Tennessee	JN182199	–	–
	TENN61384	USA: Great Smoky Mountains National Park, Tennessee	JN182200	–	–
*M.pelianthina* (Fr.) Quél.	108b	Italy: Venice Museum of Natural History, Venice	JF908379	–	–
* M. pelianthina *	108f	Italy: Venice Museum of Natural History, Venice	JF908380	–	–
	CBH164	Denmark: Jutland, Paderup Mose	FN394548	–	–
*M.pseudocorticola* Kühner	124a	Italy: Venice Museum of Natural History, Venice	JF908386	–	–
*M.pura* (Pers.) P. Kumm.	HMJAU 43121	Liaoning: Ant Ridge, Dandong City	MK309793	–	–
* M. pura *	HMJAU 43179	Heilongjiang: Shengshan National Nature Reserve	MK309794	–	–
	TENN65043	USA: Great Smoky Mountains National Park, Tennessee	JN182202	–	–
*M.rosea* Gramberg	CBH409	Germany: Baden-Württemberg, Schwarzwald	FN394551	–	–
* M. rosea *	TL12409	Denmark: Jutland, Skivum Nørrekrat	FN394557	–	–
*M.rosella* (Fr.) P. Kumm.	938a	Italy: Venice Museum of Natural History, Venice	JF908488	–	–
* M. rosella *	Champ-21	JGI MycoCosm database	KX449424	–	–
*M.seminau* A.L.C.Chew&Desjardin	ACL136	Malaysia: Ulu Gombak, Selangor	KF537250	–	–
* M. seminau *	ACL308	Malaysia: Ulu Gombak, Selangor	KF537252	–	–
*M.silvae-nigrae* Maas Geest.&Schwöbel	515	Italy: Venice Museum of Natural History, Venice	JF908452	–	–
* M. silvae-nigrae *	CC 13-12	USA: Great Smoky Mountains National Park	KF359604	–	–
*M.substylobates* T.Bau & Q.Na	HMJAU 43418, **Type**	Guangdong: Chebaling National Nature Reserve, Shaoguan City	MH216189	–	–
* M. substylobates *	HMJAU 43444	Guangxi Zhuang Autonomous Region: Nonggang National Nature Reserve, Chongzuo City	MH216190	–	–
*M.supina* (Fr.) P. Kumm.	128a	Italy: Venice Museum of Natural History, Venice	JF908388	–	–
*M.tenerrima* Maas Geest.	HMJAU 43646	Chongqing: Huangshui Town	MK309795	–	MK629340
* M. tenerrima *	HMJAU 43816	Tibet: Bomi County, Nyingchi City	MK309796	MK629364	–
*M.zephirus* (Fr.) P. Kumm.	CBS 270.48	Netherlands: Microbial Biological Resource Centres	MH856339	–	–
* M. zephirus *	CBS 273.48	Netherlands: Microbial Biological Resource Centres	MH856341	–	–

### Sequence alignment and phylogenetic analysis

A dataset, comprising sequences for the ITS + nLSU + SSU region from 96 accessions with taxonomic coverage of Europe, North America, Australia, Africa and Asia, was compiled and analysed. Sequences for 32 accessions were downloaded from GenBank and 64 newly generated sequences obtained in this study were aligned and adjusted manually using BioEdit 7.0.4.1 and Clustal X (Thompson et al. 1997; Hall 1999). The alignment was deposited with TreeBase (submission ID, 24326; study accession URL: http://purl.org/phylo/treebase/phylows/study/TB2:S24326). *Infundibulicybegibba* were chosen as the outgroup. The aligned dataset consisted of 817 ITS, 1530 nLSU and 620 SSU nucleotide sites (including gaps). The best-fit evolutionary model was identified using Modeltest 2.3 for each of the ITS, nLSU and SSU data partitions for Bayesian Inference (BI), which was implemented with MrBayes 3.2.6 (Ronquist and Huelsenbeck 2003; Nylander 2004). Markov chain Monte Carlo (MCMC) chains were run for one million generations, sampling every 100^th^ generation until the critical value for the topological convergence diagnostic was less than 0.01 (Ronquist and Huelsenbeck 2003). Maximum Likelihood (ML) analysis was performed in raxmlGUI 1.5b1, with a rapid bootstrapping algorithm involving 1,000 replicates (Stamatakis et al. 2004). Topology support values greater than 75% bootstrap support (ML) 0.95 and Bayesian posterior probabilities (BPP) are shown at each branch node.

## Results

### Phylogeny

Sect. Amparoina (Clade 5) formed a distinct clade separated from sect. Sacchariferae (Clade 4), sect. Calodontes (Clade 3), sect. Supinaae (Clade 2) and sect. Fragilipedes (Clade 1), as a sister group to all other clades within the ingroup with high statistical support (ML ≥ 75%, BPP ≥ 1.00) and should be elevated to section level.

**Figure 1.**
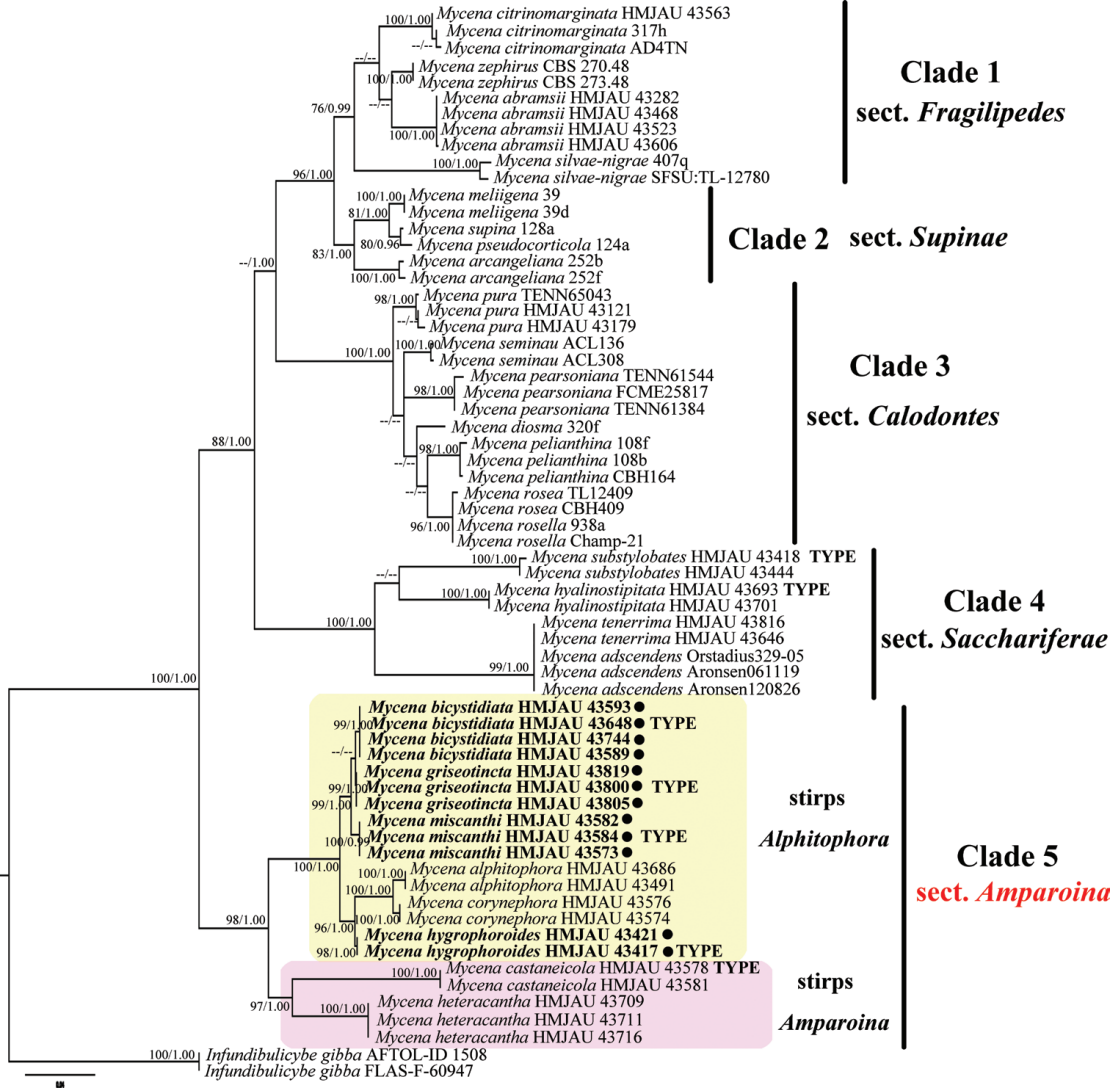
Maximum Likelihood and Bayesian tree concatenated ITS+nLSU+SSU dataset (ML ≥ 75%, BPP ≥ 0.95 are indicated). The tree is rooted with *Infundibulicybegibba*. The new species are marked by ●.

Phylogenetic reconstructions obtained using BI and ML showed similar topologies. The best-scoring Maximum Likelihood (ML) tree was selected as a representative phylogeny (Fig. 1). The optimal evolutionary model for the 5.8S and nLSU partition were lset nst = 6, rates = invgamma and prset statefreqpr = dirichlet (1,1,1,1) and SSU was lset nst = 6, rates = gamma and prset statefreqpr = dirichlet (1,1,1,1). The phylogenetic tree contained six clades, five including species of *Mycena*. The latter clade was nested within the clades of *Mycena* species. Each of the five clades of *Mycena* species corresponded with a taxonomic section, circumscribed from morphological characters, with high statistical support (ML ≥ 75%, BPP ≥ 0.95).

Samples of the four new species were placed in separate monophyletic lineages, each with high statistical support (*M.bicystidiatum*, ML = 99%, BPP = 1.00; *M.griseotincta*, ML = 99%, BPP = 1.00; *M.hygrophoroides*, ML = 98%, BPP = 0.99; *M.miscanthi*, ML = 100%, BPP = 1.00; Fig. 1). The phylogenetic tree resolved a strongly supported stirps *Alphitophora* comprising these species along with *M.alphitophora* (Berk.) Sacc., *M.corynephora* Maas Geest. in Clade 5 with ML = 100%, BPP = 1.00. Then stirps *Amparoina*, also located in Clade 5 as sister group with stirps *Alphitophora*, formed a monophyletic lineage with high statistical support in accordance with a basal disc in morphology. The distinction of the new taxa from the closely related species, *M.alphitophora* and *M.corynephora*, was also supported.

### Taxonomy

#### Key to species of sect. Amparoina and sect. Sacchariferae in China

**Table:** 

1	Basal disc present, cherocytes absent, acanthocysts present, caulocystidia smooth or with few spines	**(sect. Sacchariferae) 2**
–	Basal disc present or absent, cherocytes present or absent, acanthocysts present, caulocystidia spinulose	**(sect. Amparoina) 5**
2	Pileus grey-black	*** M. anoectochila ***
–	Pileus white	**3**
3	Caulocystidia irregularly shaped	*** M. substylobates ***
–	Caulocystidia fusiform	**4**
4	Cheilocystidia fusiform with spines in the middle part	*** M. tenerrima ***
–	Cheilocystidia sphaeropedunculate with spines overall	*** M. hyalinostipitata ***
5	Basal disc and cherocytes present	(**stirps *Amparoina*) 6**
–	Basal disc and cherocytes absent	(**stirps *Alphitophora*) 7**
6	Habitat on fruits of *Castanea*, pileus slightly pubescent	*** M. castaneicola ***
–	Habitat on dead wood or humus layer, pileus with bran-like covering	*** M. heteracantha ***
7	Lamellae distant, L < 10, I < 3	*** M. hygrophoroides ***
–	Lamellae normal, L > 15, I > 6	**8**
8	Basidiomata typically grey	*** M. griseotincta ***
–	Basidiomata white	**9**
9	Caulocystidia of two types, sphaeropedunculate or clavate	*** M. bicystidiata ***
–	Caulocystidia clavate	**10**
10	Basidiospores globose	*** M. corynephora ***
–	Basidiospores ellipsoid	***11***
11	Acanthocysts of one type, sphaeropedunculate	*** M. miscanthi ***
–	Acanthocysts of two types, globose or long-clavate	*** M. alphitophora ***

#### Section Amparoina

Taxon classificationFungiAgaricalesMycenaceae

T.Bau & Q.Na sect. nov.

829096

##### Diagnosis.

Pileus densely pubescent to furfuraceous. Stipe arising from a well-developed basal disc or base swollen without a basal disc. Cheilocystidia with spines. Cherocystes present or absent. Acanthocysts present and overlying universal veil. Caulocystidia densely spinulose overall, never smooth.

##### Type species.

*Mycenaspinosissima* (Singer) Desjardin

##### Etymology.

Name refers to the name of stirps *Amparoina*.

#### Mycena bicystidiata

Taxon classificationFungiAgaricalesMycenaceae

T.Bau & Q.Na sp. nov.

829097

Figs 2c–d , 3

##### Diagnosis.

Pileus furfuraceous to pruinose. Stipe without basal disc. Basidiospores small, 6.1–7.9 × 3.7–4.6 μm. Cheilocystidia clustered, sphaero-pedunculate to utriform with numerous sharp excrescences. Cherocytes absent. Acanthocysts pyriform to vesicular. Caulocystidia of two types, sphaero-pedunculate or clavate covered with conic spines. Clamps present.

##### Holotype.

CHINA. Chongqing City, Dafengbao Scenic Regions, 15 Aug 2017, Qin Na, HMJAU 43648.

##### Etymology.

Name refers to its two types of caulocystidia.

##### Description.

Pileus 2.8–5.2 mm in diam., conical when young, becoming nearly hemispherical with age, pure white all over, sulcate, translucent-striate, pruinose, furfur-like scattered, margin entire first, then nearly plane and finally fissile. Context very thin and fragile, pure white. Lamellae 0.5 mm thick, narrowly adnate, off-white, concolorous with the sides. Stipe slender, 15–28 × 0.5–1.0 mm, cylindrical, hollow, fragile, pure white, densely pruinose on the whole surface, base swollen and not forming a basal disc, hirsute. Odour and taste inconspicuous.

Basidiospores (5.6-)6.1–7.9(-8.3) × (3.5)3.7–4.6(4.9) μm, Q=1.6–2.0, ellipsoid to oblong-ellipsoid, hyaline, with drops, thin walled, amyloid. Basidia 20–26 × 6–9 μm, clavate, hyaline, 4- or 2-spored. Cheilocystidia 19–32 × 12–18 μm, clustered, sphaero-pedunculate to utriform with numerous sharp spines, thin-walled and hyaline, inamyloid. Pleurocystidia absent. Pileipellis hyphae 4–7 μm wide, weakly dextrinoid; cherocytes absent; a cutis overlaid by elements of universal veil, not in chains; acanthocysts of one type, numerous, pyriform to vesicular, 29–62 × 24–51 μm, inamyloid. Hyphae of the stipitipellis 3–14 μm wide, smooth, dextrinoid; caulocystidia abundant, of two types, utriform, sphaero-pedunculate, 21–85 × 14–66 μm or clavate, long-elliptic, 21–85 × 11–26 μm, densely and evenly spinulose overall, hyaline, thin-walled, inamyloid. Clamps present in all tissues.

##### Habit and habitat.

Solitary to scattered on rotten wood in mixed forests, Bamboos, *Cunninghamia*, *Ginkgo* and *Platycladus* forests.

##### Other specimens examined.

CHINA. Hubei Province, Enshi Tujia and Miao Autonomous Prefecture, Lichuan County, Yandongwan, 19 Jul 2017, Qin Na, HMJAU 43589; Xingdou Mountain National Nature Reserves, 20 Jul 2017, Qin Na, HMJAU 43593; Zhejiang Province, Hangzhou City, Tianmu Mountain National Nature Reserves, 4 Jul 2018, Qin Na and Tolgor Bau, HMJAU 43774.

##### Remarks.

*Mycenabicystidiata* is unique in sect. Amparoina stirps *Alphitophora* because of the two types of caulocystidia covered with conic spines. *Mycenaalphitophora*, which is the most widely distributed species of sect. Amparoina, shows the most morphological similarities to *M.bicystidiatum*; however, the former differs in forming cylindric spores (7.5–10 × 4.5–5.5 μm), sphaero-pedunculate cheilocystidia and caulocystidia that are only clavate in shape (Desjardin 1995). *Mycenadepilata* Singer is easily mistaken for *M.bicystidiata* by the stipe without a basal disc and the similar shape and size of spores and cheilocystidia, but *M.depilata* is distinguished from *M.bicystidiata* by its small basidiomata (pileus < 0.3 mm), larger spores (8.5–10 × 4.5–5.2 μm), and long-cylindrical and larger caulocystidia (30–120 × 5–20 μm) (Desjardin 1995). In contrast to *M.bicystidiata*, basidiospores of *M.corynephora*, *M.distincta* (Manim. & Leelav.) Aravind. & Manim., *M.globispora* (Manim. & Leelav.) Aravind. & Manim. and *M.yalensis* Singer are globose or broadly ellipsoid (Desjardin 1995; Aravindakshan and Manimohan 2015). The bright or dark colour on the pileus distinguishes *M.brunneospinosa* Desjardin, *M.incarnativelum* Desjardin and *M.roseotincta* Aravind. & Manim. from *M.bicystidiata* (Desjardin 1995; Aravindakshan and Manimohan 2015). In addition, *M.hemitrichialis* Singer produces caulocystidia that are only partially spinulose (Singer 1989).

**Figure 2.**
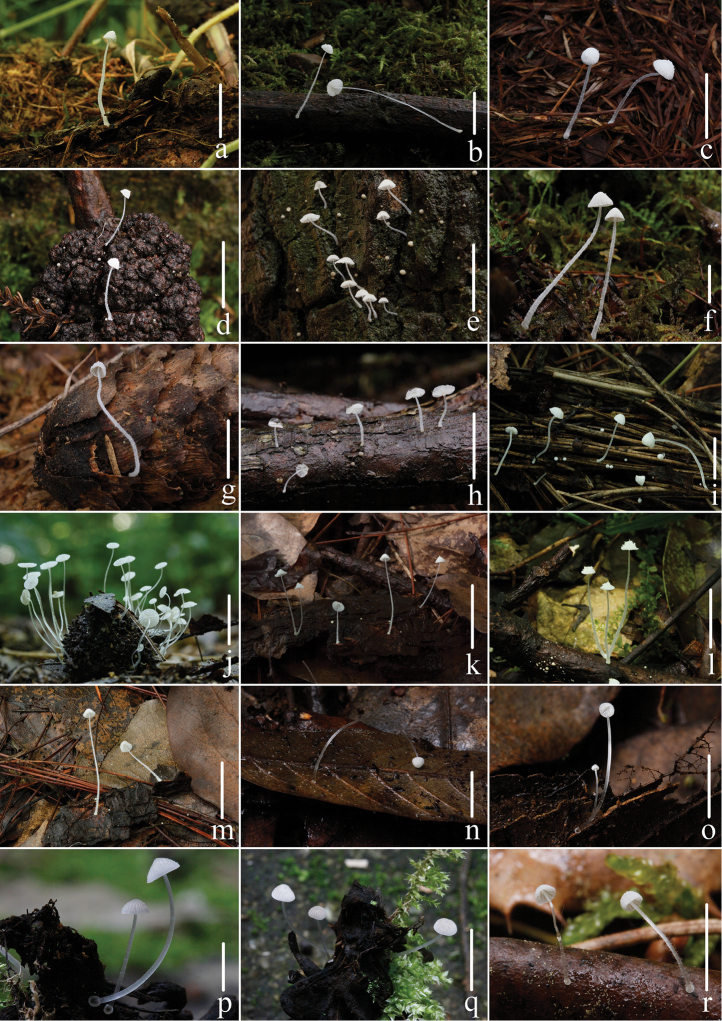
Basidiomata of sect. Amparoina species. stirps *Alphitophora*: **a–b***Mycenaalphitophora* (Berk.) Sacc. **c–d***Mycenabicystidiata* T.Bau & Q.Na **e***Mycenacorynephora* Maas Geest. **f–g***Mycenagriseotincta* T.Bau & Q.Na **h***Mycenahygroporoides* T.Bau & Q.Na **i***Mycenamiscanthi* T.Bau & Q.Na; stirps *Amparoina*: **j***Mycenacastaneicola* T.Bau & Q.Na **k–m***Mycenaheteracantha* (Singer) Desjardin. Basidiomata of sect. Saccariferae species **n–o***Mycenahyalinostipitata* T.Bau & Q.Na **p–q***Mycenasubstylobates* T.Bau & Q.Na **r***Mycenatenerrima* (Berk.) Quél. (=*Mycenaadscendens* Maas Geest.) Scale bars: 10 mm (**a–g, i–m, r**), 5 mm (**h, n–q**). Photographs **a–r** by Qin Na.

**Figure 3.**
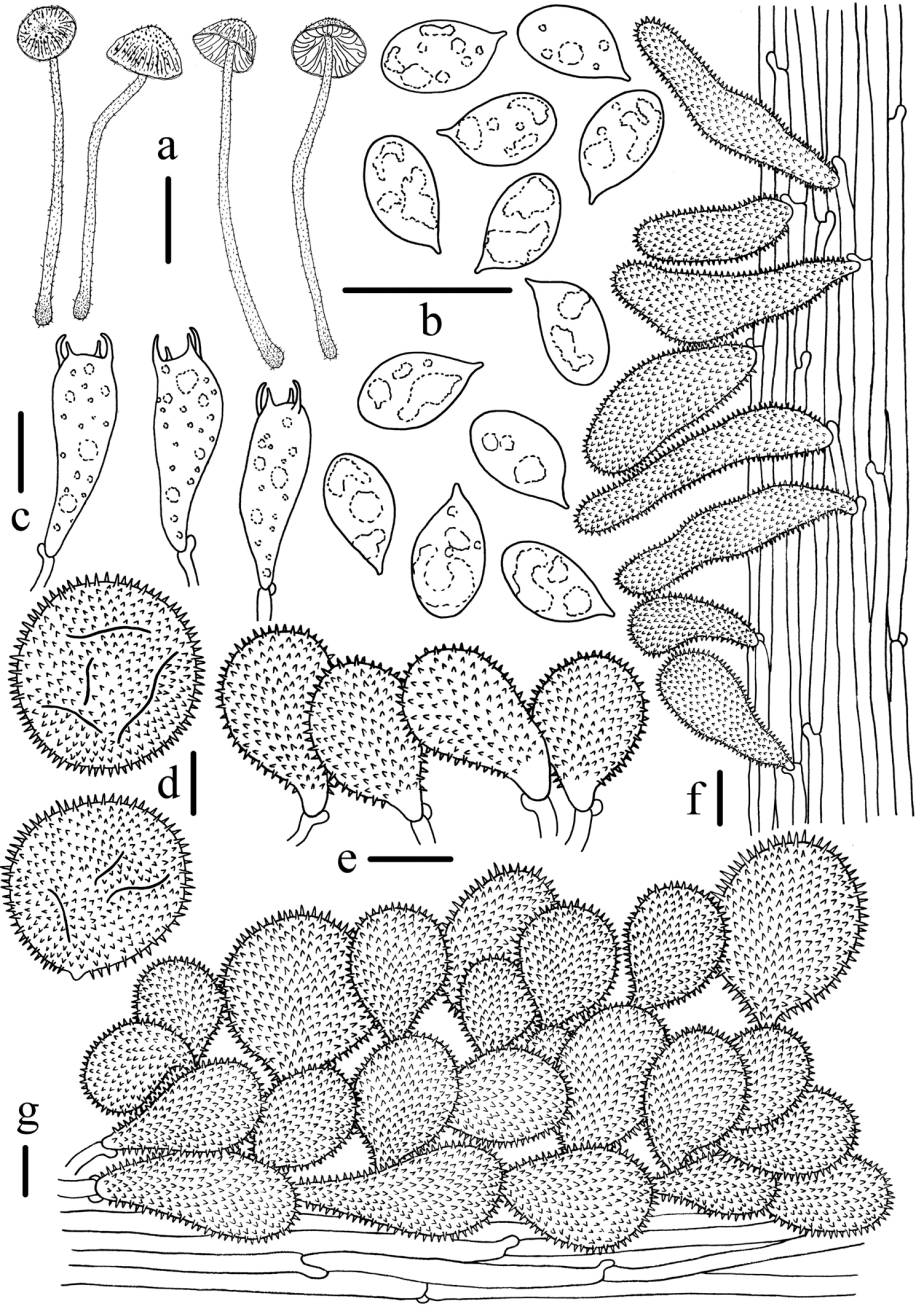
Microscopic features of *Mycenabicystidiata* (HMJAU 43648, holotype) **a** Basidiomata **b** Basidiospores **c** Basidia **d** Universal veil acanthocysts **e** Cheilocystidia **f** Caulocystidia **g** Pileipellis. Scale bars: 5 mm (**a**); 10 μm (**b–g**). Drawing by Qin Na.

#### Mycena griseotincta

Taxon classificationFungiAgaricalesMycenaceae

T.Bau & Q.Na sp. nov.

829098

Figs 2f–g , 4

##### Diagnosis.

Pileus, lamellae and stipe with greyish tint, especially when old. Stipe base swollen. Basidiospores pip-shaped. Pileipellis with two types of acanthocysts. Caulocystidia up to 200 μm long with spines.

##### Holotype.

CHINA. Yunnan Province, Diqing Tibetan Autonomous Prefecture, Shangri-La Pudacuo National Park, 14 August 2018, Qin Na, HMJAU 43800.

##### Etymology.

Name refers to the grey-tinted basidiomata.

##### Description.

Pileus 1.5–12.8 mm in diam., conical when young, campanulate with age, obtusely umbonate in the centre, translucent-striate, white, greyish-white when old (4B1), floccose, pubescent, pruinose, with crenate margin when young, then becoming nearly plane and finely torn. Context pure white, thin, fragile. Lamellae 0.2–0.5 mm thick, narrowly adnate or adnexed, pure white to slightly pale grey (4B1); edges finely torn, concolorous with the sides. Stipe 13–64 × 0.5–1.0 mm, central, terete, almost equal or slightly tapering to apex, hollow, greyish-white (5B1), pubescent or puberulous, with white, fine hairs, base swollen. Odourless, taste mild.

Basidiospores (5.6-)6.3–8.2(-8.5) × (3.5-)4.2–4.6(-5.2) μm, Q=1.5–1.9, Qav=1.7, pip-shaped, hyaline, guttulate, thin walled, amyloid. Basidia 19–23 × 7–9 μm, hyaline, clavate, 4-spored. Cheilocystidia 17–28 × 11–19 μm, oblong or clavate, with short and sharp spines, hyaline, inamyloid. Pleurocystidia absent. Pileipellis hyphae 6–10 μm wide, strongly dextrinoid; cherocytes absent; acanthocysts of two types, pyriform to vesicular, 8–22 × 7–18 μm or clavate to cylindric, 17–51 × 8–13 μm; universal veil composed of acanthocysts, globose, subglobose or sphaero-pedunculate, 28–67 × 26–58 μm, hyaline, covered with long, cylindrical excrescences or long and flexuous spinules, not in chains. Hyphae of the stipitipellis 2–7 μm wide, dextrinoid; caulocystidia abundant, clavate or long cylindrical, 77–216 × 9–11 μm, covered with densely conic spines, inamyloid. Clamps not seen.

##### Habit and habitat.

Scattered to gregarious on litter layer in *Quercus*, *Picea*, *Abies*, *Pinus* mixed forests.

##### Other specimens examined.

Yunnan Province, Diqing Tibetan Autonomous Prefecture, Shangri-La Pudacuo National Park, 15 August 2018, Qin Na, HMJAU 43805; Tibet Autonomous Region, Nyingchi City, Zhuqudeng Village, 20 August 2018, Qin Na, HMJAU 43819.

##### Remarks.

*Mycenagriseotincta* is considered a new species in sect. Amparoina stirps *Alphitophora* on account of the absence of both a basal disc and cherocytes on the pileal surface (Desjardin 1995). Five species have ellipsoid basidiospores, caulocystidia covered with excrescences and a universal veil composed of acanthocysts: *M.alphitophora*, *M.brunneospinosa*, *M.depilata*, *M.hemitrichialis* and *M.incarnativelum*. *Mycenaalphitophora* most resembles *M.griseotincta*, but the former differs in having pure white lamellae, a white and shorter stipe (< 50 mm), sphaero-pedunculate or obovoid cheilocystidia and larger spores (8.1–9.7 × 4.5–5.5 μm), as reported in the original description (Maas Geesteranus 1980, 1992b). *Mycenabrunneospinosa*, a taxon named by Desjardin (1995), is readily identified by its dull brown or purplish-brown pileus, globose acanthocysts forming chains and broadly ellipsoid spores. *Mycenaincarnativelum* is a unique species in sect. Sacchariferae, distinguished by the absence of cheilocystidia and deep pink basidiomata when young (Desjardin 1995). *Mycenadepilata* is closely allied to *M.griseotincta*, but differs in the convex pileus less than 1 mm in diameter and short and broadly clavate caulocystidia (Singer 1989). *Mycenahemitrichialis* can be mistaken for *M.griseotincta* on account of its grey or pallid pileus and ellipsoid spores, but is distinguished from *M.griseotincta* by its white stipe, free lamellae and pilose stipe forming a flattened ring of mycelium (Desjardin 1995). *Mycenacorynephora* is widely distributed worldwide and is recognised by its tiny basidiomata (pileus < 2.4 mm), absence of a basal bulb or basal disc and large globose to subglobose basidiospores, typical of stirps *Alphitophora* (Desjardin 1995; Robich 2003; Aronsen and Læssøe 2016). The same spore shape occurs in *M.yalensis* of which the holotype was collected from Argentina (Singer 1973). Aravindakshan and Manimohan (2015) reported one new species and two others newly combined in *Mycena*, collected from India. The new taxon, *M.roseotincta*, differs from *M.griseotincta* in its pink pileus and universal veil, subcylindrical spores and smaller caulocystidia (Aravindakshan and Manimohan 2015). *Mycenaglobispora* and *M.distincta* are mainly distinguished in macromorphology from *M.griseotincta* by their white basidiomata and, in micromorphology, by the globose spores and subcylindrical spores, respectively (Aravindakshan and Manimohan 2015).

**Figure 4.**
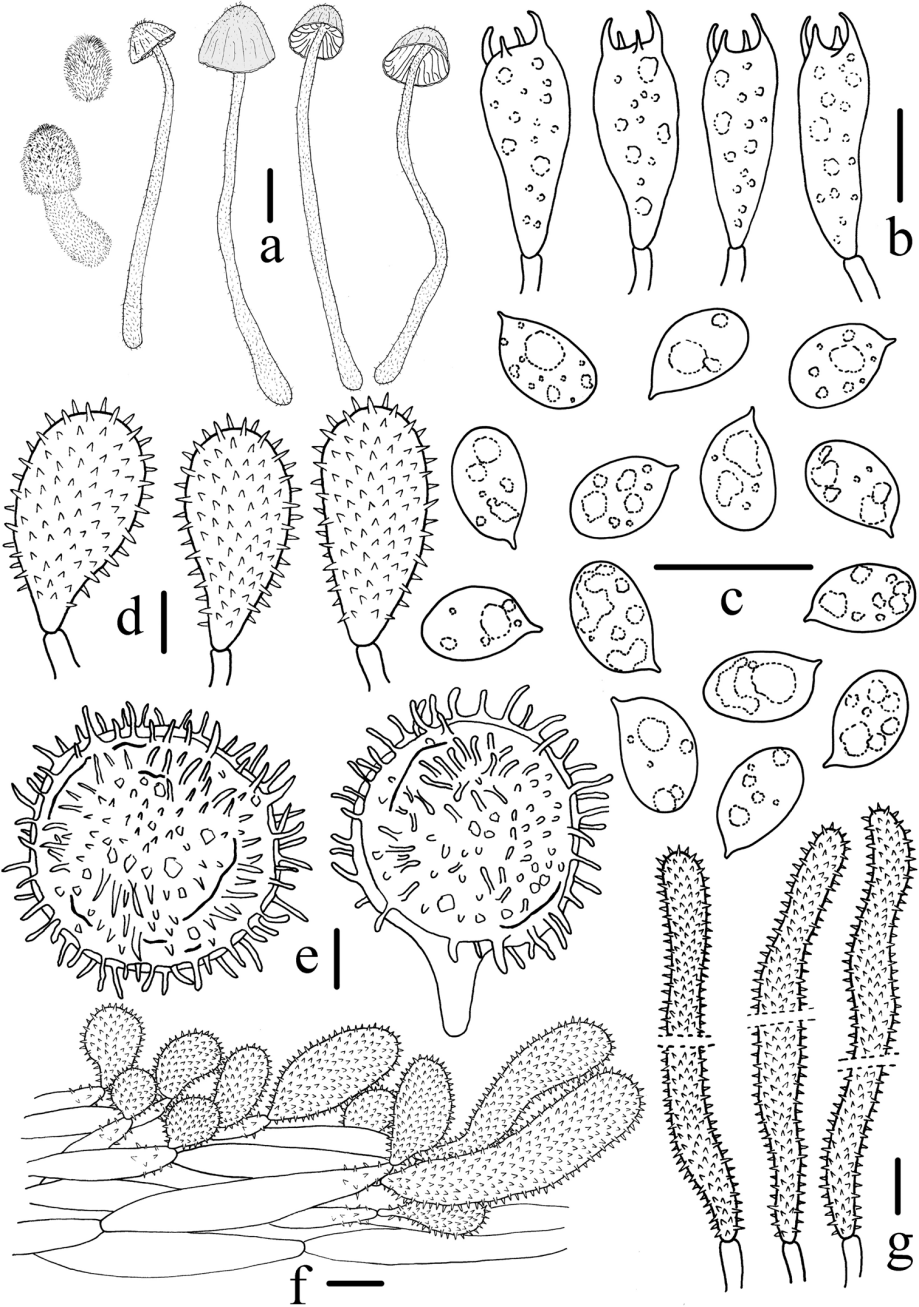
Microscopic features of *Mycenagriseotincta* (HMJAU 43800, holotype). **a** Basidiomata **b** Basidia **c** Basidiospores **d** Cheilocystidia **e** Universal veil acanthocysts **f** Pileipellis **g** Caulocystidia. Scale bars: 10 mm (**a**); 10 μm (**b–g**). Drawing by Qin Na.

#### Mycena hygrophoroides

Taxon classificationFungiAgaricalesMycenaceae

T.Bau & Q.Na sp. nov.

829099

Figs 2h , 5

##### Diagnosis.

Pileus concave with slight pruinose. Lamellae distant. Stipe with dense white fibrils and swollen base. Acanthocysts forming two types. Caulocystidia long-elliptic with conical excrescences, up to 120 μm long.

##### Holotype.

CHINA. Guangdong Province, Shaoguan City, Chebaling National Nature Reserve, 8 May 2017, Qin Na, HMJAU 43417.

##### Etymology.

Name refers to its sparse lamellae.

##### Description.

Pileus 1.5–2.5 mm in diam., campanulate to hemispherical, applanate or slightly concave at centre, white with greyish shade (6B1), shallowly sulcate, translucent-striate, slightly pruinose, pubescent. Context white, thin and very fragile. Lamellae distant, sparse, white, concolorous with the sides. Stipe 4.5–8.2 × 0.5–0.8 mm, cylindrical, hollow, fragile, pure white (5A1) with a greyish (5B1) base, covered with dense white fibrils, base swollen and not forming basal disc, hirsute. Odour and taste indistinctive.

Basidiospores (6.9-)7.2-8.9(-9.3) × (5.3-)6.4-6.7(-7.1) μm, Q=1.2–1.5, Qav=1.31, broadly-ellipsoid, hyaline in water and 5% KOH, amyloid, smooth. Basidia 15–21 × 7–9 μm, 4- or 2-spored, clavate, hyaline. Cheilocystidia 23–37 × 19–28 μm, subglobose, sphaero-pedunculate to utriform with numerous sharp spines, thin-walled and hyaline, inamyloid. Pleurocystidia absent. Pileipellis hyphae 3–9 μm wide, dextrinoid; cherocytes absent; a cutis overlaid by elements of universal veil, not in chains; acanthocysts forming two types, pyriform to vesicular, 13–29 × 11–24 μm, clavate to ovoid or obovoid, 29–42 × 14–20 μm, inamyloid. Hyphae of the stipitipellis 3–7 μm wide, smooth, dextrinoid; caulocystidia abundant, clavate, long-elliptic, 32–122 × 8–11 μm, with numbers of conical spines, inamyloid. Clamps present in all tissues.

##### Habit and habitat.

Scattered on rotten wood of coniferous trees, ex. *Cunninghamia*.

##### Other specimens examined.

Guangdong Province, Shaoguan City, Liangjiang Town, Shangxie Village, 7 May 2017, Qin Na, HMJAU 43421.

##### Remarks.

*Mycenahygrophoroides* could be considered to be a member of *Hemimycena* Singer owing to the tiny basidiomata and sparse lamellae, but the absence of a basal disc, amyloid spores and spinulose cheilocystidia, acanthocysts and caulocystidia are diagnostic characters for *M.hygrophoroides*, which should be placed in Mycenasect.Amparoina stirps *Alphitophora*. *Mycenaacanthophila* J.C.Zamora&Català, of which the holotype was collected from Spain growing on dead branches of Leguminosae, most resembles *M.hygrophoroides*, but differs in having a yellow pileus, smaller cheilocystidia (13.5–22 × 8.5–12 μm) and diverse caulocystidia (Zamora and Català 2012). *Mycenadepilata*, a species of stirps *Alphitophora*, shows some morphological similarities to *M.hygrophoroides* in possessing white and tiny basidiomata, distant lamellae (L = 7–9) and globose-pedicellate acanthocysts with hyaline contents. However, *M.depilata* differs in producing ellipsoid spores (Q = 1.64 ± 0.11), broadly clavate cheilocystidia and shorter caulocystidia (16–50 × 5–16 μm; Singer 1989). *Mycenahemitrichialis* is difficult to distinguish from *M.hygrophoroides*, but *M.hemitrichialis* has free to subfree lamellae, longer caulocystidia (100–300 × 5–15 μm) and ellipsoid spores (Singer 1989). In comparison with *M.hygrophoroides*, *M.alphitophora* and *M.distincta* have larger basidiomata and longer caulocystidia of more than 400 μm and 300 μm, respectively (Desjardin 1995; Aravindakshan and Manimohan 2015). Their noticeably pigmented pileus enables discrimination of *M.brunneospinosa*, *M.incarnativelum* and *M.roseotincta* from *M.hygrophoroides* (Desjardin 1995; Aravindakshan and Manimohan 2015). The significantly larger basidiomata and globose spores can be used to distinguish *M.corynephora*, *M.globispora* and *M.yalensis* from *M.hygrophoroides*.

**Figure 5.**
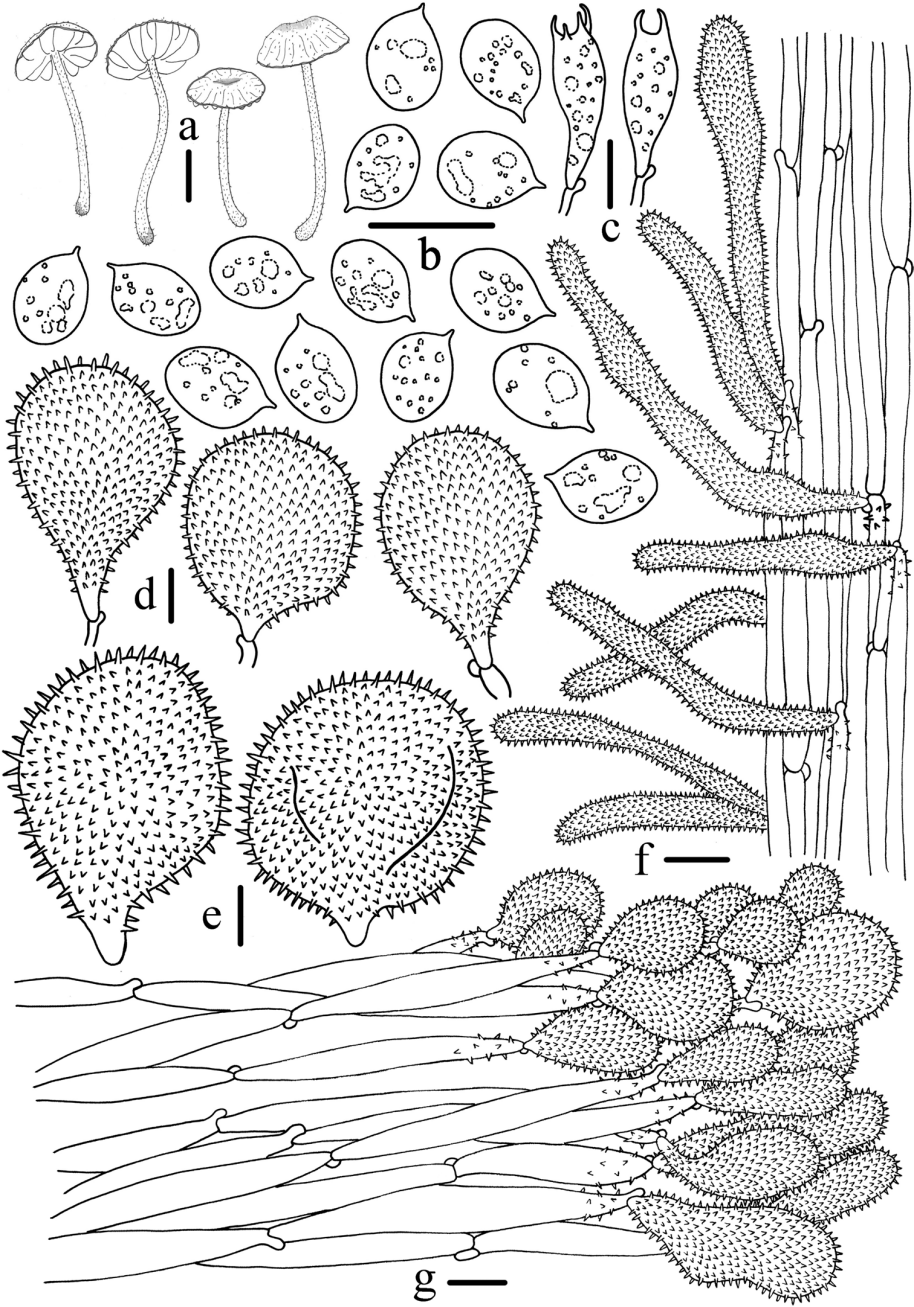
Microscopic features of *Mycenahygrophoroides* (HMJAU 43417, holotype) **a** Basidiomata **b** Basidia **c** Basidiospores **d** Cheilocystidia **e** Universal veil acanthocysts **f** Caulocystidia **g** Pileipellis. Scale bars: 2 mm (**a**); 10 μm (**b–g**). Drawing by Qin Na.

#### Mycena miscanthi

Taxon classificationFungiAgaricalesMycenaceae

T.Bau & Q.Na sp. nov.

829100

Figs 2i , 6

##### Diagnosis.

Growing on dead stem of *Miscanthus*. Pileus sparsely pruinose. Basidiospores cylindric. Cherocytes absent. Acanthocysts forming two types. Caulocystidia sphaero-pedunculate covered with spines. Clamps present.

##### Holotype.

CHINA. Henan Province: Xinyang City, Jigong Mountain, 16 Jul 2017, Qin Na and Tolgor Bau, HMJAU 43584.

##### Etymology.

Name refers to the substratum where the new species was found.

##### Description.

Pileus 3.5–7.8 mm in diam., hemispherical, broadly conical to convex, occasionally ± centrally depressed when young, sulcate, translucent-striate, pure white, pubescent to inconspicuously puberulous, margin nearly plane, undulate. Context white, thin, very fragile, about 1.0 mm thick at centre. Lamellae narrowly adnate or adnexed, off-white, concolorous with the sides. Stipe 26–38 × 0.5–1.0 mm, pure white, central, terete, hollow, equal, surface covered with slight white pubescent, base swollen but not discoid, pruinose. Odour and taste not distinctive.

Basidiospores (6.2-)6.7–8.6(-9.1) × (3.1)3.3–4.2(4.5) μm, Q=1.8–2.3, Qav=2.07, cylindric to narrow-ellipsoid, hyaline, guttulate, thin walled, amyloid. Basidia 18–24 × 6–9 μm, clavate, hyaline, 4-spored. Cheilocystidia 13–26 × 9–14 μm, abundant, lageniform, utriform or sphaero-pedunculate, with short and conical spines. Pleurocystidia absent. Pileipellis hyphae 3–8 μm wide, strongly dextrinoid; cherocytes absent; universal veil composed of acanthocysts, forming two types, pyriform, vesicular or clavate, 12–32 × 10–17 μm, inamyloid. Hyphae of the stipitipellis 2–8 μm wide, with coarse excrescences, 0.9–2.8 × 0.5–0.9 μm, strongly dextrinoid; caulocystidia abundant, elliptic, utriform, sphaero-pedunculate, 15–37 × 7–15 μm, with conical or cylindrical spines inamyloid. Clamps present in all tissues.

##### Habit and habitat.

Solitary to scattered on dead stem of *Miscanthus*.

##### Other specimens examined.

Henan Province, Xinyang City, Jinniu Mountain, 14 Jul 2017, HMJAU 43573; Xinyang City, Bolden National Forest Park, 17 July 2017, Qin Na and Tolgor Bau, HMJAU 43582.

##### Remarks.

The distinctive features of *Mycenamiscanthi* include a white, granulose pileus, a pubescent stipe without forming a basal disc, narrow-ellipsoid spores, two types of acanthocysts and growth on dead stems of *Miscanthus* species. In combination, these features support the placement of *M.miscanthi* in sect. Amparoina stirps *Alphitophora*. Similar to *M.miscanthi*, *M.alphitophora* and *M.depilata* produce pure white basidiomata, cylindric spores and sphaero-pedunculate and spinulose cheilocystidia (Desjardin 1995; Aravindakshan and Manimohan 2015). However, the two types of acanthocysts and longer caulocystidia can be used to distinguish *M.alphitophora* and *M.depilata* from *M.miscanthi* (Desjardin 1995). *Mycenahemitrichialis* is closely allied to *M.miscanthi*, but differs in producing caulocystidia up to 400 μm in length that lack spinulae or with a few spinulae in the upper half (Singer 1989). *Mycenadistincta*, which was originally described as M.alphitophoravar.distincta, was elevated to species level by Manimohan and Leelavathy (1989). It differs from *M.miscanthi* in producing broadly ellipsoid spores and caulocystidia up to 300 μm in length (Aravindakshan and Manimohan 2015). The pigmented pileus present in *M.brunneospinosa*, *M.incarnativelum* and *M.roseotincta* readily distinguishes these species from *M.miscanthi* (Desjardin 1995; Aravindakshan and Manimohan 2015). *Mycenacorynephora*, *M.globispora* and *M.yalensis* of stirps *Alphitophora* are characterised by globose to subglobose spores (Maas Geesteranus 1980; Robich 2003; Aravindakshan and Manimohan 2015; Aronsen and Læssøe 2016).

**Figure 6.**
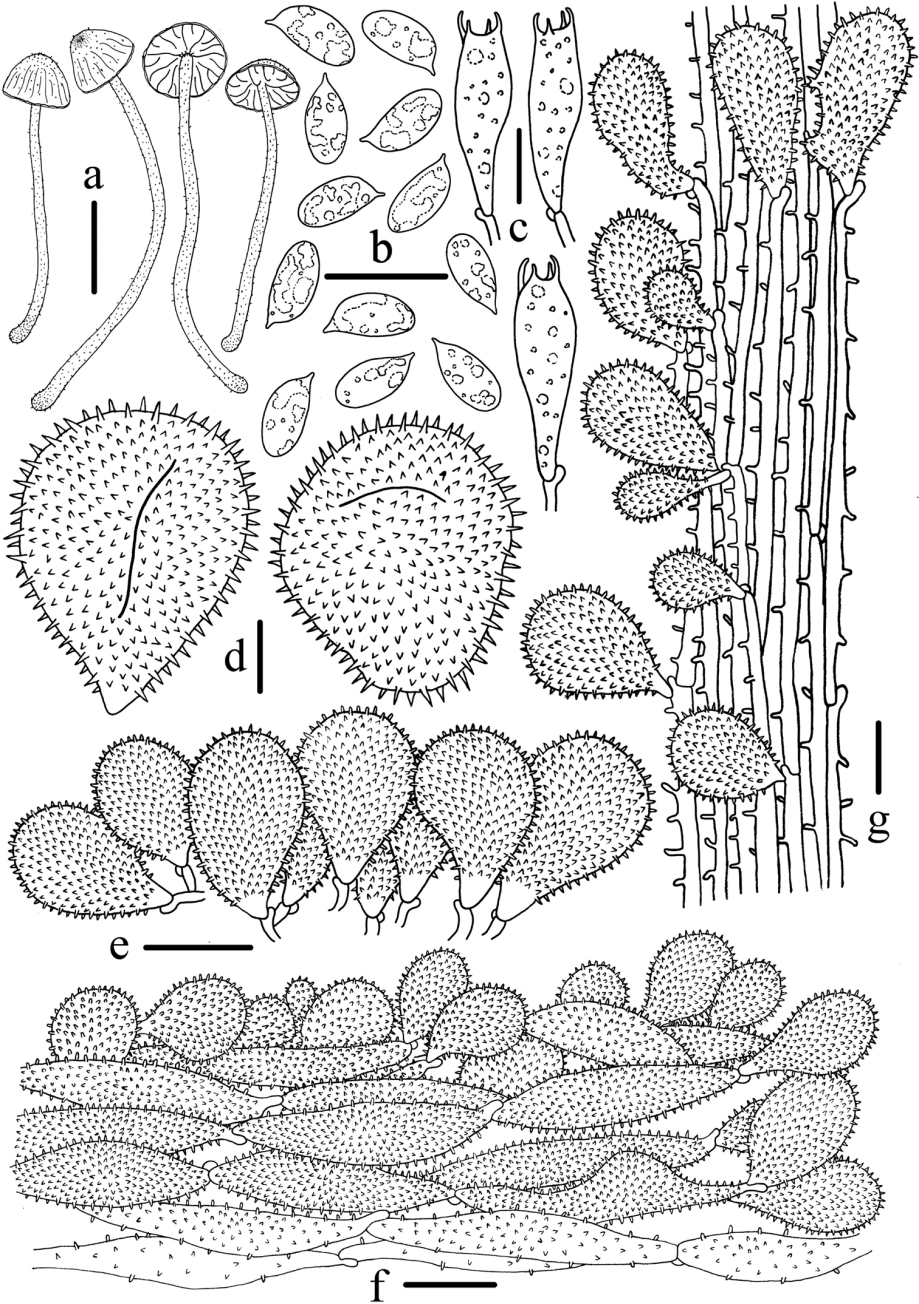
Microscopic features of *Mycenamiscanthi* (HMJAU 43584, holotype) **a** Basidiomata **b** Basidiospores **c** Basidia **d** Universal veil acanthocysts **e** Cheilocystidia **f** Pileipellis **g** Caulocystidia. Scale bars: 10 mm (**a**); 10 μm (**b–g**). Drawing by Qin Na.

## Discussion

The present phylogenetic analysis showed that sect. Amparoina formed a distinct clade independent from sect. Sacchariferae with high BPP and BS support. This finding suggests that the presence of caulocystidia with dense spines is the most important character to separate sect. Amparoina from sect. Sacchariferae. However, in the presence of a basal disc, the species of sect. Sacchariferae are similar to stirps *Amparoina* and, in the acanthocysts on the pileus sect. Amparoina stirps, resembles sect. AmparoinaSacchariferae. It can be concluded that the difference in caulocystidia can be used to distinguish sect. Amparoina and sect. Sacchariferae and the basal disc and cherocytes are the basis of an infrasectional classification of sect. Amparoina. Thus, the circumscription of sect. Sacchariferae should be revised, for which the diagnostic characters are a well-developed basal disc, cherocytes absent, pileipellis a cutis not overlaid by elements of a universal veil composed of acanthocysts and caulocystidia smooth overall.

In morphology, sect. Amparoina and sect. Sacchariferae are closely allied with sect. Polyadelphiae Singer ex Maas Geest. and sect. Basipedes (Fr.) Quél (Desjardin et al. 2003). Species of sect. Polyadelphiae lack both ornamented pileipellis elements and a stipe with a basal disc and thus differ from species classified in sect. Amparoina and sect. Sacchariferae. Section Basipedes shares the same habitat and a stipe forming a developed basal disc, but the cheilocystidia are covered with rounded and few excrescences. Morphological characters distinguish sect. Polyadelphiae and sect. Basipedes from sect. Amparoina and sect. Sacchariferae and only one ITS sequence for *M.stylobates* (Pers.) P. Kumm. (JF908439) is currently deposited in GenBank.

Morphological characters and molecular evidence support the classification of the four new Mycena species as members of sect. Amparoina stirps *Alphitophora*. The four species share the same furfuraceous or farinose pileus, swollen stipe base without a basal disc, universal veil composed of acanthocysts and absence of both cherocytes and spinose caulocystidia. *Mycenabicystidiatum* is distinguished from *M.griseotincta*, *M.hygrophoroides* and *M.miscanthi* by producing two types of caulocystidia covered with conic spines. *Mycenagriseotincta* is readily discriminated from *M.bicystidiatum*, *M.hygrophoroides* and *M.miscanthi* based on the greyish basidiomata and acanthocysts forming a universal veil with long, cylindrical excrescences. Compared with *M.bicystidiatum*, *M.griseotincta*, and *M.miscanthi*, *M.hygrophoroides* is distinct on account of the sparse lamellae and broadly ellipsoid basidiospores. *Mycenamiscanthi* differ from *M.bicystidiatum*, *M.griseotincta* and *M.hygrophoroides* in growing on stems of *Miscanthus* and, in addition, the basidiospores are narrow ellipsoid.

It is worth mentioning that the placement of *M.echinocephala* (G.F. Atk.) Desjardin and *M.cylindrospora* A.H. Sm. remains unclear. The species are tentatively placed in stirps *Alphitophora* because of the lack of a basal disc on the stipe, but their caulocystidia are extraordinary in being smooth, terminated by a spinulose apex or smooth with an amorphous apex (Atkinson 1902; Smith 1947; Desjardin 1993). Both species show obvious differences to the four newly described taxa. Furthermore, *M.cryptomeriicola* Imazeki & Toki is distinctive in producing inamyloid spores and a basal disc, which is unusual for specimens of sect. Sacchariferae from Japan (Imazeki and Toki 1995). An additional unusual species, *M.minya* Grgur., which lacks caulocystidia, was reported from Australia (Grgurinovic 2003). No species similar in morphology to *M.cryptomeriicola* and *M.minya* are classified in sect. Sacchariferae, so the two species are tentatively accepted in sect. Sacchariferae.

## Supplementary Material

XML Treatment for Amparoina

XML Treatment for Mycena bicystidiata

XML Treatment for Mycena griseotincta

XML Treatment for Mycena hygrophoroides

XML Treatment for Mycena miscanthi

## References

[B1] Aravindakshan DM Manimohan P (2015) Mycenas of Kerala. SporePrint Books, Calicut, India. 10.13140/RG.2.1.2116.4003

[B2] Aronsen A Læssøe T (2016) The Genus *Mycena* s.l. Fungi of Northern Europe Vol. 5. Narayana Press, Gylling, Denmark.

[B3] Atkinson GF (1902) Three new genera of higher fungi. Botanical Gazette 34: 36–43. 10.1086/328258

[B4] Cortéspérez A Ramírezguillén F Guzmán G (2015) Nuevos registros de *Mycena* sección *Sacchariferae* (Basidiomycota) para México. Revista Mexicana de Micologia 41: 79–87

[B5] Desjardin DE (1993) Notes on *Mycenacylindrospora* and *Eomycenellaechinocephala*. Mycologia, 85(3): 509–513. 10.2307/3760711

[B6] Desjardin DE (1995) A preliminary accounting of the worldwide members of Mycenasect.Sacchariferae. Bibliotheca Mycologica 159: 1–89.

[B7] Desjardin DE Boonpratuang T Hywel-Jones N (2003) New spinose species of *Mycena* in sections *Basipedes* and *Polyadelphia* from Thailand. Fungal Diversity 12: 7–17.

[B8] Grgurinovic CA (2003) The genus *Mycena* in south-eastern Australia. Fungal Diversity Press, Canberra, Australia.

[B9] Guo SX Fan L Cao WQ Xu JT Xiao PG (1997) *Mycenaanoectochila* sp. nov. isolated from mycorrhizal roots of *Anoectochilusroxburghii* from Xishuangbanna, China. Mycologia 89: 952–954. 10.2307/3761116

[B10] Hall TA (1999) BioEdit: a user-friendly biological sequence alignment editor and analysis program for Windows 95/98/NT. Nucleic Acids Symposium Series 41: 95–98.

[B11] Hopple JS Vilgalys R (1999) Phylogenetic relationships in the mushroom genus *Coprinus* and dark-spored allies based on sequence data from the nuclear gene coding for the large ribosomal subunit RNA: divergent domains, outgroups, and monophyly. Molecular Phylogenetics & Evolution 13(1): 1–19. 10.1006/mpev.1999.0634 10508535

[B12] Horak E (2005) Röhrlinge und Blätterpilze in Europa: Bestimmungsschlüssel für Polyporales (pp), Boletales, Agaricales, Russulales. Elsevier, Spektrum Akad Verlag.

[B13] Imazeki R Toki S (1955) Contributions to the knowledge of Japanese Agaricales. Bulletin of the Government Forest Experimental Station Meguro, 79: 1–14.

[B14] Kirk PM Cannon PE Minter DW Stalpers JA (2008) Dictionary of the Fungi (10 edition). Wallingford: CABI International.

[B15] Kornerup A Wanscher JHK (1978) The Methuen Handbook of Colour. Eyre Methuen, London.

[B16] Kühner R (1938) Le genre *Mycena* (Fries). Encyclopédie Mycologique X. P. Lechevalier 10: 1–710.

[B17] Li Y Li TH Yang ZL Bau T Dai YC (2015) Atlas of Chinese Macrofungal Resources. Central Chinese Farmer Press, Zhengzhou, China.

[B18] Lodge DJ (1988) Three new *Mycena* species (Basidiomycota: Tricholomataceae) from Puerto Rico. Transactions of the British Mycological Society 91(1): 109–1﻿﻿16. 10.1016/s0007-1536(88)80011-1

[B19] Maas Geesteranus RA (1980) Studies in Mycenas-15. Persoonia 11: 93–120.

[B20] Maas Geesteranus RA (1983) Conspectus of the Mycenas of the Northern Hemisphere-1, Sections *Sacchariferae*, *Basipedes*, *Bulbosae*, *Clavulares*, *Exiguae*, and *Longisetae*. Proceedings van de Koninklijke Nederlandse Akademie van Wetenschappen (Ser C), Amsterdam, North-Holland 86: 401–421.

[B21] Maas Geesteranus RA (1992a) Mycenas of the Northern Hemisphere I. Studies in Mycenas and other papers. Proceedings van de Koninklijke Nederlandse Akademie van Wetenschappen, Amsterdam, North-Holland.

[B22] Maas Geesteranus RA (1992b) Mycenas of the Northern Hemisphere II. Studies in Mycenas and other papers. Proceedings van de Koninklijke Nederlandse Akademie van Wetenschappen, Amsterdam, North-Holland.

[B23] Maas Geesteranus RA de Meijer AAR (1997) Mycenae Paranaenses. Proc K Ned Akad Wet, Amsterdam, North-Holland.

[B24] Maas Geesteranus RA de Meijer AAR (1998) Further Mycenas from the state of Paraná, Brazil. Persoonia 17(1): 29–46.

[B25] Na Q Bau T (2018) New species of *Mycena* (Mycenaceae, Agaricales) with colored lamellae and three new species records from China. Phytotaxa 361(3): 266–278. 10.11646/phytotaxa.361.3.2

[B26] Na Q Bau T (2019) MycenasectionSacchariferae: three new species with basal discs from China. Mycological Progress 18: 483–493. 10.1007/s11557-018-1456-8

[B27] Nealel B (2009) Two intimately co-occurring species of MycenasectionSacchariferae in south-west Australia. Mycotaxon 108(4): 159–174. 10.5248/108.159

[B28] Nylander J (2004) MrModeltest v2. Program distributed by the author. Evolutionary Biology Centre, Uppsala University, Uppsala.

[B29] Perry BA (2002) A taxonomic investigation of *Mycena* in California. Doctoral dissertation, San Francisco State University, California, USA.

[B30] Persoon CH (1797) Tentamen dispositionis methodicae fungorum in classes ordines, genera et familias. Lipsiae. 10.5962/bhl.title.42674

[B31] Robich G (2003) *Mycena* d’Europa. Associazione Micologica Bresadola, Trento, Italy.

[B32] Robich G Hausknecht A (2009) *Mycenabhuglooi*, a new species of section Sacchariferae (Agaricales, Tricholomataceae) from Mauritius (Africa). Österr Z Pilzk 18: 7–14.

[B33] Robich G (2016) *Mycena* d’Europa Volume 2. Associazione Micologica Bresadola, Trento, Italy.

[B34] Ronquist F Huelsenbeck JP (2003) MrBayes 3: Bayesian phylogenetic inference under mixed models. Bioinformatics 19: 1572–1574. 10.1093/bioinformatics/btg180 12912839

[B35] Singer R (1958) New genera of fungi VIII. Notes concerning the sections of the genus *Marasmius* Fr. Mycologia 50: 103–110. 10.1080/00275514.1958.12024714

[B36] Singer R (1973) Diagnose fungorum novorum Agaricalium III. Sydowia 15: 45–83.

[B37] Singer R (1976) Amparoinaceae and Montagneaceae. Revue de Mycologie 40: 57–64.

[B38] Singer R (1989) New taxa and new combinations of Agaricales (Diagnose fungorum novorum Agaricalium IV). Fieldiana 21: 1–133. 10.5962/bhl.title.2537

[B39] Smith AH (1947) North American species of *Mycena*. University Michigan Press, Ann Arbor, Michigan.

[B40] Stamatakis A Ludwig T Meier H (2004) RAxML-III: a fast program for maximum likelyhood-based inference of large phylogenetic trees. Bioinformatics 21(4): 456–463. 10.1093/bioinformatics/bti191 15608047

[B41] Takahashi H (1999) *Mycenaauricoma*, a new species of *Mycena*, section Radiatae, from Japan, and *Mycenaspinosissima*, a new record in Japan. Mycoscience 40(1): 73–80. 10.1007/bf02465677

[B42] Tanaka I Hongo T (2003) Two new records of Mycenasect.Sacchariferae from Japan and type study of *Mycenacryptomeriicola* (sect. Sacchariferae). Mycoscience 44(6): 421–424. 10.1007/s10267-003-0134-z

[B43] Thompson JD Gibson TJ Plewniak F (1997) The Clustal-X windows interface: flexible strategies for multiple sequence alignment aided by quality analysis tools. Nucleic Acids Research 63: 215–228. 10.1093/nar/25.24.4876 PMC147148 9396791

[B44] Ward E Gray RM (2010) Generation of a ribosomal DNA probe by PCR and its use in identification of fungi within the Gaeumannomyces-*Phialophora* complex. Plant Pathology 41(6): 730–736. 10.1111/j.1365-3059.1992.tb02556.x

[B45] White TJ Bruns T Lee S Taylor J (1990) Amplification and direct sequencing of fungal ribosomal RNA genes for phylogenetics. In: Innis MA Gelfand DH Sninsky JJ White TJ (Eds) PCR protocols: a guide to methods and applications.Academic, San Diego, 315–322. 10.1016/b978-0-12-372180-8.50042-1

[B46] Zamora JC Català S (2013) A new species of Mycenasect.Sacchariferae from the Iberian cushion-shaped Genisteae. Mycotaxon 122(4): 361–368. 10.5248/122.361

